# Commencements

**Published:** 1899-05

**Authors:** 


### EDITORIAL.

### Commencements.

This is about the time of year many dental colleges have completed their work of training the members of their senior class, and has come to the time of sending them out to test their ability, in the way of practice.

Within a few weeks all the colleges will have completed this work. This is a time of greater anxiety and solicitude to the student than any other time of his preparatory course.

At the time of graduation a large proportion of graduates are uncertain as to the future, and the question comes to them "what will be the next move in my career?" This question ought to come to every student at or soon after the beginning of his preparatory course, and ought to be a stimulus to put forth every effort and energy for the accomplishment of the highest results.

Usually those who pursue this course are best equipped and find the most ready opportunity for the exercise of professional skill.

There will perhaps be a larger number of graduates than ever before.

The June number will contain a list of graduates of nearly all the colleges of the country.

